# Hotel smoking policies and their implementation: a survey of California hotel managers

**DOI:** 10.1186/s12971-017-0147-6

**Published:** 2017-10-30

**Authors:** Joy M. Zakarian, Penelope J. E. Quintana, Carl H. Winston, Georg E. Matt

**Affiliations:** 1 San Diego State University Research Foundation, 9245 Sky Park Court, Suite 225, San Diego, California, 92123 USA; 2 Graduate School of Public Health, San Diego State University, San Diego, California, 92182 USA; 3 L. Robert Payne School of Hospitality & Tourism Management, San Diego State University, San Diego, California, 92182 USA; 4 Department of Psychology, San Diego State University, San Diego, California, 92182 USA

**Keywords:** Secondhand smoke, Thirdhand smoke, Smoking policy, Hospitality industry, Smoke-free hotels

## Abstract

**Background:**

Most states in the U.S. permit hotels to allow smoking in some guest rooms, and only five (Indiana, Michigan, North Dakota, Vermont, and Wisconsin) require that all hotel and motel rooms be 100% smoke-free (State and local 100% smokefree hotel and motel guest room laws enacted as of July 3, 2017). Little is known, however, about how hotels’ smoking policies have been implemented. This study examined hotels’ smoking policies and their implementation.

**Methods:**

A telephone survey of a random sample of 383 California hotel managers was conducted.

**Results:**

Overall, 60.6% of hotels reported that smoking was prohibited in all guest rooms, and 4.7% reported that smoking was prohibited everywhere on their property. While California law permitted smoking in up to 65% of guest rooms, only 6.9% of rooms were reported as smoking-permitted. Over 90% of hotels had smoking rooms scattered among nonsmoking rooms, and about half of the smoking hotels reported that guests requesting either smoking or nonsmoking rooms were sometimes assigned to the other room type. When guests smoked in nonsmoking rooms fees could be substantial, but were often uncollected.

**Conclusions:**

Hotel smoking policies and their implementation fall short of protecting nonsmoking guests and workers from exposure to secondhand and thirdhand smoke. Complete indoor smoking bans for all hotels are needed to close existing loopholes. Nonsmokers who wish to protect themselves from exposure to tobacco smoke should avoid hotels that permit smoking and instead stay in completely smoke-free hotels.

## Background

Most states in the U.S. permit hotels to allow smoking in some guest rooms, and only five (Indiana, Michigan, North Dakota, Vermont, and Wisconsin) require that all hotel and motel rooms be 100% smoke-free [1]. For instance, while California Labor Code has prohibited smoking of tobacco products in almost all indoor workplaces since 1995, the hospitality industry was granted several notable exceptions. Specifically, smoking has been permitted in up to 65% of guest rooms, 25–50% of lobby areas (depending on total square feet), and meeting and banquet rooms, except while food or beverage functions are taking place [2].

When the hospitality exceptions were adopted, they represented a compromise between tobacco control advocates concerned about the health consequences of secondhand smoke (SHS) exposure and hospitality industry concerns about the financial impact of a total smoking ban. Since then, attitudes among Californians have moved overwhelmingly towards expanding tobacco control efforts, and new state and local ordinances now ban tobacco use in parks, beaches, private cars when children are present, college campuses, and an increasing number of multiunit housing buildings [3–6]. Moreover, a growing body of research demonstrates that partial indoor smoking bans do not protect nonsmokers from SHS exposure, [7] and that indoor tobacco use leads to the accumulation of residual tobacco smoke toxicants, also known as thirdhand smoke (THS), and subsequent exposure of nonsmokers to these toxicants long after cigarettes have been smoked [7–10]. See Lewinson & Bryant for a discussion of tobacco use and exposure in extended stay hotels [11].

The trend in tobacco use in hospitality venues has paralleled the decrease in the general population. A 2016 hospitality industry survey found that overall, 85% of U.S. hotels have 100% nonsmoking rooms, ranging from 99% for luxury properties to 62% for midscale chains and 42% for economy hotels [12]. Consistent with changes in attitudes toward tobacco use in the general population, this was a substantial increase in smoke-free properties compared to 63% in 2014 and 2012, 56% in 2010 and 38% in 2008. Data provided by the American Automobile Association indicate that in 2011, California had 1575 completely smoke-free hotels, motels, and other lodgings, a 51% increase from 2008 [13].

Little is known, however, about how the smoking exceptions have been implemented in hotels that do permit smoking. For instance, in what proportion of hotel guest rooms are guests permitted to smoke? How are guests informed about smoking policies? How do hotels respond to violations of smoking policies? And, how satisfied are hotel guests with current smoking policies? This paper addresses these issues, presenting findings from a telephone survey of a random sample of California hotel managers.

## Methods

### Sample

A listing of 1000 randomly selected lodging properties in California was obtained from the largest hospitality trade association in the state (i.e., California Hotel and Lodging Association) in December 2008. Research assistants attempted to contact the owner, general manager, or manager on duty for 533 properties listed. Of these, 404 were determined to be California properties that were in business and open to the public. Completed telephone surveys were obtained for 292 properties, with 91 partially completed and 21 declining to participate. Surveys were conducted from March 2009 to February 2010. Respondents were not compensated for their participation.

### Measures

#### Telephone survey

Respondents were asked about the number and location of smoking-permitted and nonsmoking guest rooms, smoking policies in other hotel areas, communication of smoking policies to guests, violations of smoking policies, and smoking policy changes under consideration. The mean time to complete the survey questions was 19.9 (95% CI [19.0, 20.9]) minutes. Surveys were audio recorded, with respondents’ permission, for ongoing training and quality control.

#### Website review

Research assistants examined the websites of the participating properties for statements about their smoking policies and whether or not the websites allowed users to reserve designated nonsmoking and smoking-permitted rooms.

#### Hotel classification

Research assistants classified hotels that received 2 or fewer stars on Expedia.com as “budget”, those with 2.5 or 3 star hotels as “midscale”, and those with more than 3 stars as “upscale”. Hotels that were not listed on Expedia.com were classified using available information obtained from the hotel’s website or via telephone query regarding the property and amenities.

### Statistical analyses

Data were analyzed with SPSS v. 22, [14] with 95% confidence intervals for proportions computed following methods developed by Newcomb [15]. To control for non-normal distributions and heterogeneous error variances of quantitative variables, logarithmic transformations were applied and geometric means are reported.

## Results

### Hotel sample

Of the 383 properties for which survey data were obtained, 57% were hotels, 24% motels, 14% bed & breakfast properties, and 5% vacation rentals. In reporting the following results, all properties are referred to as “hotels”. Overall, 182 (47.5%) properties were classified as budget, 147 (38.4%) midscale, and 47 (12.3%) upscale. Seven properties were unable to be classified due to incomplete data.

### Prevalence of 100% smoke-free properties

Overall, 4.7% (95% CI [2.8, 7.7]) of respondents reported that smoking was not permitted anywhere on their hotel property, including guest rooms, hallways, the lobby, banquet or meeting rooms, or outdoors: 1.4% (95% CI [0.07, 8.5]) of budget properties, 4.4% (95% CI [1.8, 9.7]) of midscale properties, and 9.2% (95% CI [4.6, 17.1]) of upscale properties. These differences were not statistically significant (χ^2^ [2, *N* = 307] = 5.45, *p* = 0.065).

### Prevalence of hotels with 100% nonsmoking guest rooms, by hotel classification

Overall, 60.6% (95% CI [55.5, 65.5]) of respondents reported that smoking was not permitted in any of their guest rooms: 46.2% (95% CI [38.8, 53.7]) of budget properties, 72.8% (95% CI [64.7, 79.6]) of midscale properties, and 83.0% (95% CI [68.7, 91.9]) of upscale properties. These differences were statistically significant (χ^2^ [2, *N* = 376] = 35.04, *p* < .001).

### Prevalence of smoking-permitted rooms

**Fig. 1:**
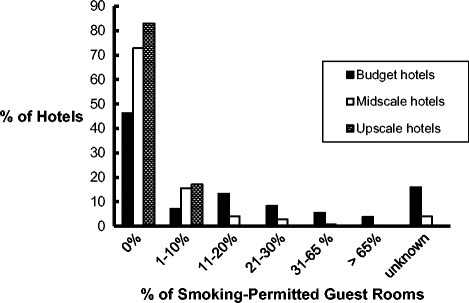
Proportion of Hotel Guest Rooms that are Smoking-Permitted. Note. All hotels with an “unknown” % reported that they do have smoking rooms, but did not report the proportion

Among the 27,798 total guest rooms in the hotels responding to the survey, 1906 (6.9%) were reported as smoking-permitted (i.e., “smoking rooms”). Among the hotels with smoking rooms, the proportion of smoking rooms varied from 1% to 100%, with a mean of 14.0% (95% CI [11.73, 16.73]) (median = 14.3%) (Fig. 1). Seven hotels (4.6% of all surveyed and 10.1% of smoking hotels) reported that smoking was permitted in more than 65% of their rooms. All of these were budget properties, four of which reported that smoking was permitted in all (100%) of their rooms.

### Prevalence of other smoking-permitted hotel areas

Hotels with smoking-permitted guest rooms (i.e., smoking hotels) were more likely than hotels without smoking-permitted guest rooms (i.e., nonsmoking hotels) to allow smoking in outdoor hallways or corridors outside of their nonsmoking rooms as well as everywhere outdoors. Nonsmoking hotels were more likely than smoking hotels to prohibit smoking in all indoor locations and to prohibit outdoor smoking or to allow it only in designated smoking areas (Table 1). Of the smoking hotels, 90.2% (95% CI [82.3, 94.9]) offered outdoor ashtrays and 2% (95% CI [0.3, 7.6]) made them available upon request; compared to 83.7% (95% CI [77.4, 88.6]) and 1.1% (95% CI [0.2, 4.3]) respectively of nonsmoking hotels.

**Table 1:** Smoking-permitted hotel locations in hotels with and without smoking-permitted guest rooms

Locations where smoking is permitted	Nonsmoking hotels (do not offer smoking-permitted guest rooms)			Smoking hotels (offer smoking-permitted guest rooms			*p*-value
	n	%	95% CI	n	%	95% CI	
Nowhere indoors	175	93.1	88.2, 96.1	0	0.0	0, 3.1	<.001
Indoor hallways outside of nonsmoking guest rooms	8	7.1	3.3, 13.9	6	13.3	5.5, 27.5	.212
Outdoor hallways outside of nonsmoking guest rooms	33	53.2	40.2, 65.8	42	77.8	64.1, 87.5	.006
Hotel lobby	4	2.2	0.7, 6.0	3	2.9	0.7, 9.0	.720
Banquet or meeting rooms	1	1.1	0.1, 6.8	2	4.5	0.1, 16.7	.203
Nowhere outdoors	17	9.1	5.5, 14.4	0	0.0	0, 4.4	.002
Only in designated outdoor areas	91	48.7	41.3, 56.0	38	36.5	27.5, 46.6	.046
Everywhere outdoors	79	42.2	35.1, 49.7	66	63.5	53.5, 72.5	.001

Note. n-sizes varied depending on if a hotel had indoor or outdoor hallways and whether or not a hotel had a lobby, banquet, or meeting rooms

### Communication of smoking policies to hotel guests

About two thirds of hotels surveyed (69.9%; 95% CI [64.7, 74.6]) included a statement about their smoking policy on their website. Of the hotels with online reservation systems and that offered nonsmoking rooms, 85.6% (95% CI [80.7, 89.5]) of the websites allowed a reservation for a specified nonsmoking room; of those that offered smoking rooms, 81.2% (95% CI [71.9, 88.0]) of the websites allowed a reservation for a specified smoking room. However, 11.1% (95% CI [7.4, 16.1]) and 29.3% (95% CI [20.0, 40.5]) of these websites stated that the reservation of a nonsmoking or smoking room, respectively, would be treated as a preference only and not guaranteed.

Respondents were asked how guests are informed at the hotel that they are not allowed to smoke in nonsmoking rooms. The most common methods endorsed were to inform guests verbally at check-in (71.0%; 95% CI [65.4, 76.1]) or to state the policy in the registration (69.5%; 95% CI [63.8, 74.7]). Over half of the properties told guests when they made a telephone reservation (57.7%; 95% CI [51.8, 63.4]) and reminded guests in an email (56.2%; 95% CI [50.2, 62.0]). About a third of the properties had a sign on the front desk or registration counter (34.4%; 95% CI [29.0, 40.1]) or elsewhere in the lobby area (26.5%; 95% CI [21.7, 30.0]). Only 4.1% (95% CI [2.2, 7.2]) of hotels reported that they did not do anything to inform guests about their smoking policy.

Table 2 shows the reported locations of signage identifying nonsmoking and smoking rooms. The most common signage reported for both types of rooms was signs located on or next to the outside of guest room doors.

**Table 2:** Location of signage identifying nonsmoking and smoking-permitted guest rooms

Signage location	Nonsmoking rooms			Smoking rooms		
	n	%	95% CI	n	%	95% CI
On or next to the outside of room doors	161	54.9	49.1, 60.7	45	41.3	32.1, 51.1
Room tables	105	36.0	30.5, 41.8	23	21.1	14.1, 30.2
On or next to the inside of room doors	90	31.0	25.8, 36.8	22	20.4	13.5, 29.4
In-room brochure or collateral	140	49.5	43.5, 55.4	23	22.1	14.8, 31.5
Hallways outside rooms	55	18.9	14.7, 24.0	13	11.9	6.8, 19.9
Nightstands	49	16.9	12.9, 21.8	17	15.6	9.6, 24.1
Other location in rooms	33	11.4	8.1, 15.7	3	2.8	0.7, 8.5
Televisions	20	6.9	4.4, 10.6	4	3.7	1.2, 9.7
Key tags	18	6.2	3.8, 9.8	1	0.9	0.1, 5.8
Bathrooms	14	4.8	2.8, 8.2	4	3.7	1.2, 9.7
Trash cans	4	1.4	0.4, 3.7	0	0	0, 4.2
Beds	2	0.7	0.1, 2.7	1	0.9	0.1, 5.8
None present	59	20.1	15.7, 25.2	18	16.5	10.3, 25.1

### Protections for nonsmoking hotel guests

#### Location of hotel rooms

Only 9.2% (95% CI [4.7, 16.6]) of multiple floor hotels had smoking rooms located on a separate floor. That is, the vast majority of hotels (> 90%) had smoking rooms scattered among nonsmoking rooms. Few hotels (29.2%; 95% CI [21.0, 39.0])) located nonsmoking rooms on a floor that was not directly above smoking rooms.

#### Room assignment

About half (49.5%; 95% CI [39.7, 59.4]) of the smoking hotels reported that guests who requested nonsmoking rooms were never assigned to smoking rooms, with a reported incidence of 0 to 300 times in the past year (mean = 8.52; 95% CI [5.49, 12.98]). Similarly, about half (52.1%; 95% CI [46.2, 57.9]) of the smoking hotels with nonsmoking rooms reported that guests who requested smoking rooms were never assigned to nonsmoking rooms, with a reported incidence of 1 to 480 occasions in the past year (mean = 10.7; 95% CI [8.2, 13.8]).

### Violations of hotel smoking policies

#### Frequency of detected smoking in nonsmoking rooms

Of the smoking hotels, 91.7% (95% CI [83.8, 96.1] reported that employees had detected that guests had smoked in nonsmoking rooms in the past year, as compared to 80.4% (95% CI [73.7, 85.8]) of nonsmoking hotels. Of the smoking hotels, 62.5% (95% CI [52.0, 72.0]) reported it had occurred less than once a month on average, 18.8% (95% CI [11.8, 28.3]) reported it had occurred more than once a month and less than once a week, and 10.4% (95% CI [5.4, 18.8]) reported once a week or more frequently. Of the nonsmoking hotels, 52.0% (95% CI [44.4, 59.4]) reported it had occurred less than once a month on average, 22.9% (95% CI [17.1, 29.9]) reported more than once a month and less than once a week, and 5.6% (95% CI [2.9, 10.3]) reported once a week or more (χ^2^ [3, *N* = 275] = 8.79, *p* = 0.32). Respondents reported that hotel employees had detected that guests had smoked in nonsmoking rooms a mean of 6.52 (95% CI [4.85, 8.66]) times in the past year at smoking hotels (range 0 to 144 times), and a mean of 4.53 (95% CI [3.55, 5.71] times at nonsmoking hotels (range 0 to 365).

#### Means of detection of smoking occurrence

The primary means of detection were a combination of smell and visual evidence such as cigarette ashes and butts left by guests in the trash cans, toilets, or throughout a hotel room (48.7%; 95% CI [42.8, 54.8]) or smell of smoke only (40.9%; 95% CI [35.1, 46.9]), with 7.5% (95% CI [4.8, 11.4]) reporting that other guests had complained or notified hotel staff that a guest was smoking in a nonsmoking room and 2.9% (95% CI [1.3, 5.8]) detecting based on visual evidence only.

#### Actions taken upon detecting smoking in nonsmoking rooms

Respondents were asked what hotel employees did if guests smoked in nonsmoking rooms. The most common response was that management, usually the housekeeping supervisor or front desk, was notified (81.4%; 95% CI [76.1, 85.8]). Some reported that guests were asked to stop smoking (55.4%; 95% CI [49.2, 61.4]), were evicted (6.0%; 95% CI [3.6, 9.7]), placed on a “do not rent” list (5.9%; 95% CI [3.6, 9.7]), forced to move to a smoking room (4.8%; 95% CI [2.7, 8.3]), or given an option of moving (1.5%; 95% CI [0.5, 4.0]). Only one respondent reported that their corporate office was notified.

#### Fees for smoking in nonsmoking rooms

Of respondents from nonsmoking hotels, 80.5% (95% CI [74.0, 85.8]) said their hotel charged an extra fee or fine if guests smoked in nonsmoking rooms; versus 72.6% (95% CI [63.0, 80.6]) of respondents from smoking hotels (χ^2^ [1, *N* = 296] = 2.44, *p* = .118). The reported fee ranged from $20 to $1600, with a mean of $168 (95% CI [155, 182]) (median = $200); 2.7% charged $20 to $45, 9.4% charged $50 to $75, 30.3% charged $100 to $150, 47.8% charged $175 to $250, 6.7% charged $300 to $475, 2.7% charged $500, and one hotel charged $1600. Of the hotels that charged a fee, 81.6% (95% CI [75.6, 86.4]) stated the fee in the registration. Only 60.9% (95% CI [53.3, 68.0]) of respondents from hotels that charged a fee said they were always successful in collecting it, with 17.3% successful 77% to 99% of the time, 4.5% successful 60% to 75% of the time, 10% successful half the time, and 7.3% of hotels successful less than half the time. Hotels had charged the fee from 0 to 200 times in the past year, with a mean of 3.4 (95% CI [2.74, 4.09]) times (median = 3.5).

#### Additional special cleaning after guests smoke in nonsmoking rooms

Table 3 shows the proportion of hotels that always or sometimes performed different types of special cleaning of nonsmoking rooms when smoking was detected. The most commonly reported types of special cleaning were shampooing the carpet, keeping the room vacant, cleaning the drapes, upholstered furniture, or walls, and using an ozone machine or deodorizing spray. Of the hotels that kept the room vacant, over half (54%) did so for longer than one day. The range was from 2 h to 8.5 days, with a mean of 35.9 (95% CI [32.6, 39.4]) hours. The mean additional time to perform special cleaning of nonsmoking rooms that have been smoked in was 2.2 h (95% CI [1.8, 2.7]), with a range of 0 to 84 h.

**Table 3:** Additional special cleaning done after guests smoke in a nonsmoking room

	*Always* done if a guest smokes in a nonsmoking room			*Sometimes* done if a guest smokes in a nonsmoking room			*Routinely* done whether or not anyone smokes in the room		
	n	*%*	95% CI	n	%	95% CI	n	%	95% CI
Clean drapes	137	56.6	50.1, 62.9	64	26.4	21.1, 32.6	22	9.1	5.9, 13.6
Shampoo carpet	137	54.4	48.0, 60.6	83	32.9	27.2, 39.2	22	8.7	5.7, 13.1
Use deodorizing spray	121	47.3	41.1, 53.6	35	13.7	9.8, 18.6	71	27.7	22.4, 33.7
Clean upholstered furniture	110	46.0	39.6, 52.6	62	25.9	20.6, 32.1	39	16.3	12.0, 21.8
Use ozone machine	119	46.7	40.5, 53.0	46	18.0	13.6, 23.4	9	3.5	1.7, 6.8
Wash bedspread	108	42.7	36.6, 49.1	12	4.7	2.6, 8.4	132	52.2	45.8, 58.4
Clean walls	100	39.1	33.1, 45.4	62	24.2	19.2, 30.0	44	17.2	12.9, 22.5
Keep room vacant	99	38.8	32.9, 45.1	116	45.5	39.3, 51.8	2	0.8	0.1, 3.1
Clean air conditioning vents	77	37.4	30.8, 44.4	22	10.7	7.0, 15.9	84	40.8	34.1, 47.8
Replace air filters	71	33.6	27.4, 40.5	29	13.7	9.5, 19.3	84	39.8	33.2, 46.8
Clean other furniture	54	20.9	16.2, 26.5	20	7.8	4.9, 11.9	179	69.4	63.3, 74.9
Wash or change all bedding	52	20.2	15.6, 25.8	6	2.3	1.0, 5.3	197	76.7	70.9, 81.6
Use carpet fresheners	45	18.3	13.8, 23.8	38	15.4	11.3, 20.7	27	11.0	7.5, 15.7
Replace furniture	2	0.8	0.1, 3.2	50	20.0	15.3, 25.6	2	0.8	0.1, 3.2
Wash or change all towels	27	10.6	7.2, 15.2	3	1.2	0.3, 3.7	223	87.5	82.6, 91.1

### Complaints about smoking

Respondents were asked how often their hotel received complaints about smoking in the past year. The most commonly endorsed response was “a few times a year” (39.2%; 95% CI [33.6 45.1]), with 37.1% (95% CI [31.6, 43.0]) reporting “never”, 18.2% (95% CI [14.1, 23.2]) “a few times a month”, and 5.5% (95% CI [3.3, 9.0]) “a few times a week”. The reported frequency of guest complaints showed a positive statistically significant correlation with the reported prevalence of hotel staff having detected that guests had smoked in nonsmoking rooms (*r* = .346, *p* < .001). There were no significant differences in overall frequency of complaints for smoking vs. nonsmoking hotels (χ^2^ [3, *N* = 291] = 1.36, *p* = .716). However, 48.6% (95% CI [38.8, 58.5]) of respondents from smoking hotels reported that they had received complaints that a guest room smelled like smoke, compared to 35.7% (95% CI [28.9, 43.1]) of respondents from nonsmoking hotels (χ^2^ [1, *N* = 290] = 4.63, *p* = .031). Nonsmoking hotels were more likely to receive complaints about outdoor smoking (21.3%; 95% CI [15.8, 28.1]) than were smoking hotels (11.4%; 95% CI [6.3, 19.5]) (χ^2^ [1, *N* = 288] = 4.47, *p* = .034. Complaints about smoke drifting into guest rooms were reported by 36.2% (95% CI [29.4, 43.6]) of nonsmoking hotels and 35.2% (95% CI [26.3, 45.2]) of smoking hotels, and complaints about smoking near the hotel entrance were reported by 16.8% (95% CI [11.8, 23.1]) of nonsmoking hotels and 8.6% (95% CI [4.2, 16.1]) of smoking hotels. These differences were not statistically significant.

### Smoking policy changes under consideration

Almost three times as many respondents from smoking hotels (21.6%; 95% CI [14.3, 31.0]) reported that their hotel was considering changes to their smoking rules, compared to respondents from nonsmoking hotels (8.0%; 95% CI [4.7, 13.1]), (χ^2^ [1, *N* = 289] = 10.85, *p* = .001). Of the 22 smoking hotels that were considering changes, 17 were considering eliminating their smoking rooms, 3 were considering reducing the number of smoking rooms, one was considering instituting a fee for smoking in nonsmoking rooms and also eliminating their smoking rooms, and one was considering adding a statement to their registration card to notify guests of their $150 fee for smoking in nonsmoking rooms. Of the 15 nonsmoking hotels that were considering changes, 5 were considering making their entire property nonsmoking, 5 were considering restrictions on outdoor smoking, 2 were considering instituting or increasing a fee for smoking in nonsmoking rooms, and 3 were considering more signage about their smoking policy and/or fee. No properties were considering increasing the number of smoking rooms or relaxing or rescinding already enacted nonsmoking policies.

## Discussion

### The majority of California hotels are nonsmoking and only a small proportion of rooms are designated as smoking-permitted

Although California labor code allowed hotels to designate up to 65% of hotel guest rooms as smoking permitted, the majority (61%) of California hotels surveyed reported to have all nonsmoking rooms. Our survey results indicate that smoking was permitted in only 7% of California hotel rooms overall, and in hotels that offered smoking rooms, an average of 14% of rooms were designated as smoking. These results are similar to national data from the same time period showing that 56% of hotels had 100% nonsmoking rooms and 9% of rooms overall were smoking-permitted [12]. None of the hotels surveyed indicated plans to increase the number of smoking rooms or to turn a nonsmoking hotel into a smoking hotel. Instead, 21.7% of hotels with smoking rooms reported plans to become 100% smoke-free or to decrease the number of rooms in which smoking was permitted.

### Poor implementation and enforcement of smoking policies

Our findings indicate that existing smoke-free policies provide exceptions that make the policies difficult to implement and are ineffective for protecting nonsmokers from exposure to secondhand and thirdhand smoke. First and foremost, in most hotels that participated in our survey, smoking rooms were not located on separate floors, but were intermingled with nonsmoking rooms. This permits tobacco smoke to migrate from the smoking rooms to hallways and nonsmoking rooms, leading to SHS exposure of nonsmokers and to the accumulation of THS in nonsmoking rooms and hallways. In addition, while hotels may have strict rules about not smoking in specified rooms, the rules are less clear and often more permissible about smoking in hallways and outdoor areas from where tobacco smoke can enter guest rooms through doors, windows, and ventilation.

Our findings also show that it is not uncommon for nonsmokers to be assigned to smoking rooms and for smokers to be assigned to nonsmoking rooms. While this is an efficient use of vacant hotel rooms, the practice is ill-advised based on what is now known about the accumulation of THS compounds and the exposure of nonsmokers to THS. Specifically, smokers are likely to pollute nonsmoking hotel rooms through off-gassing and direct contact from their clothing, hair, and skin even if they do not smoke in the rooms. At the same time, nonsmokers who stay in smoking rooms will be unknowingly exposed to the THS pollutants that have accumulated in the rooms when previous guests smoked.

Most hotel managers said that their hotels used at least one method to inform guests about nonsmoking rooms (e.g., upon booking, verbally at check-in, in the registration, or through signage in the hotel). However, based on our results, over 40% of guests who reserve rooms by telephone were reportedly not told that they were reserving a nonsmoking room, and 29% who reserved online did not see an explicit message about the hotel’s smoking policy. A small but potentially significant proportion of guests (13–17%) who made online reservations were not able to reserve a specific room type (i.e., smoking or nonsmoking). And of those online reservation systems that did accept reservations for a specified smoking or nonsmoking room, this was noted as a “request” only for as many as a third of these systems. If hotels’ smoking policies were clearly conveyed to all guests upon making reservations, smokers might be less likely to inadvertently stay in a nonsmoking room where they may smoke due to convenience and habit. The respondents also indicated that signage regarding smoking policy was absent in 20.1% of nonsmoking rooms and 16.5% of smoking rooms. In summary, while the number of hotels with smoking rooms and the proportion of smoking rooms in these hotels continue to decline, the design and implementation of the existing policies fail to protect nonsmokers from tobacco smoke exposure. This is because the chemical and physical properties of tobacco smoke do not allow pollutants to be constrained to a designated space, [16–18] and because hotels’ economic considerations demand flexibility in the assignment of rooms during times of high occupancy rates.

### Increased costs and inadvertent health risks from smoking clean-up efforts

While our survey did not determine the costs for cleaning and repairs after a guest has smoked, our findings show that hotels incur significant expenses over and above regular cleaning and maintenance. Among the most important expenses are likely to be shampooing carpets, cleaning drapes, cleaning walls, and keeping rooms vacant.

Among the most common methods used to address lingering tobacco odor after smoking is the use of an “ozone machine”. These machines are designed to emit the oxidant ozone in the indoor air so that it may initiate a chemical reaction that transforms odorant chemical compounds of SHS and THS into odorless compounds. Recent research, however, indicates that this practice may create more harm than benefits because some of the newly created compounds may be more toxic than the initial compounds [19–21]. This practice should be carefully scrutinized, and if used at all, be used only if the rooms are subsequently sufficiently ventilated and cleaned to remove any newly generated oxidized compounds.

### Hospitality smoking regulations continue to have important loopholes

Our previous research has shown that a partial hotel smoking ban did not protect nonsmoking hotel rooms from tobacco smoke pollution nor nonsmokers staying in these rooms from exposure to it [10]. In nonsmoking rooms of hotels that also offered smoking rooms, mean levels of surface and air nicotine and air 3-ethenylpyridine (3EP), as well as nicotine on the hands of nonsmokers who stayed overnight in the rooms, were higher than in nonsmoking rooms of hotels that did not offer smoking rooms. Nonsmokers who wish to protect themselves from exposure to tobacco smoke should avoid hotels that permit smoking and instead stay in completely smoke-free hotels.

Publicly available documents show that the tobacco industry has provided funds to and worked with more than 65 hospitality groups in the U.S., including several in California [22]. Dearlove and colleagues have shown that Phillip Morris began promoting an “accommodation” strategy to the hospitality industry in 1989, recommending the use of ventilation systems and harmony between smokers and nonsmokers by accommodating both, as opposed to smoking restrictions [22]. Other tobacco companies have adopted similar programs. Despite evidence to the contrary, the tobacco industry spread misinformation to the hospitality industry that they would suffer financially by banning smoking. It is unknown to what extent the tobacco industry continues to influence hotels to provide smoking rooms. However, our survey results show that about 40% of California hotels put their nonsmoking guests at risk for exposure to tobacco pollution by offering smoking rooms. Lower-income nonsmokers may be at highest risk, as our survey data as well as national data show that the proportion of hotels with smoking rooms is highest in budget properties and intermediate in midscale properties compared to upscale properties [12]. A small proportion of budget properties responding to our survey reported that they did not even have nonsmoking rooms, or that greater than 65% of their rooms were designated for smoking, and therefore appeared to be in violation of California law.

## Conclusions

### Recommendations and directions for future research

Our previous study found that nonsmoking signage in rental cars was associated with lower levels of THS pollutants in dust and air [23]. All hotels should identify nonsmoking rooms with signage to reduce the likelihood of a smoker unknowingly smoking in a nonsmoking room. To help protect nonsmokers, hotels also need to honor a room’s designation as smoking or nonsmoking, allow guests to select their room type when reserving online or by telephone, and assign guests to their selected room type on every occasion. While a majority of hotels said they imposed a fine for smoking in nonsmoking rooms, almost half of these said that they did not always collect the fee. More rigorous enforcement could lead to increased compliance. In about half of the instances where hotel employees detected unauthorized smoking, visual evidence was lacking and smell or other guests’ complaints were the only indicators. Real-time monitoring for air particulate matter could potentially notify hotel staff when smoking occurs and allow early intervention. The majority of California hotels surveyed reported employing a variety of additional cleaning activities when a guest was found to have smoked in a nonsmoking room. Our survey did not investigate the costs associated with these clean-up efforts. Future studies might examine the costs compared to the income generated from renting smoking rooms.

Given the overwhelming public support for smoke-free indoor environments and existing trends in the hospitality industry, the time may have come to promulgate a complete indoor smoking ban for all hotels. The California legislature has recently taken a step in this direction by revising the California labor code to remove the exceptions provided for hotel lobbies and meeting rooms and to decrease the proportion of guest rooms in which smoking is permitted from 65% to 20%, effective June, 2016. In lieu of a complete indoor smoking ban, hotels could be required to restrict smoking rooms to separate floors or even better separate buildings with independent ventilation systems from nonsmoking rooms. However, these protections will not apply to housekeeping staff and other workers who will be exposed to SHS and THS smoke pollution as long as smoking is permitted in their workplace. Pearson and colleagues reported that 22% of hotel housekeeping staff surveyed were bothered by smoke in their work area and 74% preferred to work in a smoke-free environment [24]. Future studies might further explore these concerns. Additional research is needed to better understand how housekeeping workers are exposed to THS in the workplace. Complete indoor smoking bans are the best solution for protecting hotel guests and workers from the harmful effects of tobacco toxicants.

A smoke-free certification for hotel rooms and other hospitality venues (e.g., Airbnb, VRBO) would help consumers to make informed decisions about staying in a tobacco-polluted environment, make tobacco use an explicit and verifiable component of the valuation of hotel rooms, and create a financial incentive in support of smoking bans. A smoke-free certification might also contribute to reducing tobacco use and increasing smoking bans in hospitality venues, where public policies are difficult to introduce and monitor. Future studies are recommended to test such certification services. Future studies might also investigate the proportion of smoke-free hotels and hotel rooms as well as hotels’ smoking policy implementation and enforcement in regions and countries with different tobacco norms, public policies, and smoking prevalence than California.

## Abbreviations

SHS: Secondhand smoke
THS: Thirdhand smoke
